# Segmentation of the fascia lata and reproducible quantification of intermuscular adipose tissue (IMAT) of the thigh

**DOI:** 10.1007/s10334-020-00878-w

**Published:** 2020-08-06

**Authors:** Oliver Chaudry, Andreas Friedberger, Alexandra Grimm, Michael Uder, Armin Michael Nagel, Wolfgang Kemmler, Klaus Engelke

**Affiliations:** 1 Department of Medicine 3, Friedrich-Alexander-Universität Erlangen-Nürnberg and University Hospital Erlangen, Ulmenweg 18, 91054 Erlangen, Germany; 2 Institute of Medical Physics, Friedrich-Alexander-Universität Erlangen-Nürnberg, Henkestrasse 91, 91052 Erlangen, Germany; 3 Institute of Radiology, Friedrich-Alexander-Universität Erlangen-Nürnberg and University Hospital Erlangen, Maximiliansplatz 3, 91054 Erlangen, Germany

**Keywords:** MRI, Fascia lata, Adipose tissue

## Abstract

**Objective:**

To develop a precise semi-automated segmentation of the fascia lata (FL) of the thigh to quantify IMAT volume in T_1_w MR images and fat fraction (FF) in Dixon MR images.

**Materials and methods:**

A multi-step segmentation approach was developed to identify fibrous structures of the FL and combining them into a closed 3D surface. 23 healthy young men with low and 50 elderly sarcopenic men with moderate levels of IMAT were measured by T_1_w and 6pt Dixon MRI at 3T. 20 datasets were used to determine reanalysis precision errors. IMAT volume was compared using the new FL segmentation versus an easier to segment but less accurate, tightly fitting envelope of the thigh muscle ensemble.

**Results:**

The segmentation was successfully applied to all 73 datasets and took about 7 min per 28 slices. In particular, in elderly subjects, it includes a large amount of adipose tissue below the FL typically not accounted for in other segmentation approaches. Inter- and intra-operator RMS-CVs were 0.33% and 0.14%, respectively, for IMAT volume and 0.04% and 0.02%, respectively, for FF_MT_.

**Discussion:**

The FL segmentation is an important step to quantify IMAT with high precision and may be useful to investigate effects of aging and treatment on changes of IMAT and FF. ClinicalTrials.gov identifier NCT2857660, August 5, 2016.

**Trial registration:**

ClinicalTrials.gov identifier NCT2857660, August 5, 2016.

## Introduction

Quantification of muscle properties such as volume and fat infiltration as well as the amount and distribution of adipose tissue (AT), is of increasing interest in diseases or conditions such as obesity, osteoporosis, rheumatoid arthritis and sarcopenia [1]. In particular, visceral and subcutaneous adipose tissue (SAT) of the abdomen and intermuscular adipose tissue (IMAT) of the thigh and tibia have been investigated [2, 3]. In addition to muscle volume, newer imaging methods provide quantification of muscle density (CT) [4] and fat fraction, i.e. multiecho chemical-shift-encoded MRI [5]. In this study, 6-point Dixon MRI was used [6].

However, it remains unclear, which parameter is most relevant. IMAT has been widely used as a semi-quantitative measure by physicians for diagnosis and treatment monitoring in neuromuscular diseases such as muscular dystrophy [7–9]. Based on visual inspection of IMAT within the deep fascia, also known as the fascia lata (FL) in CT or standard T1 weighted MR images they assign scores for fat accumulation and distribution [10]. However, a more quantitative analysis requires a stricter definition of the parameters and the specific anatomical location. Usually, the differentiation between tissues requires an accurate segmentation of the fascia lata of the thigh. Especially in CT with relatively low soft tissue contrast, this can be a challenging task [11, 12].

This study focusses on MR imaging of the mid-thigh. The FL separates SAT from muscles surrounded by perimuscular adipose tissue. About 80% of adipose tissue consists of lipids [13] stored in adipocytes, which can also be found among muscle fibres [14]. In T_1_ weighted images, larger agglomerations of adipocytes within muscles appear hypointens contrasting the darker muscle tissue. According to the traditional definition, these agglomerations together with the perimuscular AT forms IMAT [2, 15]. Other contributions of intramuscular adipose tissue—smaller agglomerations of adipocytes as well as intracellular lipids—cannot be detected on T_1_ weighted images, but contribute to fat fraction (FF) that can be quantified by Dixon MRI [16].

Whether the quantification of IMAT volume and its distribution or the measurement of intramuscular FF is more relevant is still an unanswered question. One reason is the rather difficult segmentation of the FL, a thin layer of fibrous tissue often difficult to identify on single MR images and due to lower soft tissue contrast even more difficult to identify on CT images [17]. Thus, instead of segmenting the FL, several authors have simply used a tight envelope of the muscle ensemble of the thigh defining the VOI for the quantification of IMAT [18–20]. However, this is problematic in elderly subjects, which typically show increased amounts of perimuscular adipose tissue between the FL and the muscle surfaces [21, 22].

Thus the primary aim of this study was the development of a novel semi-automated 3D segmentation method of the FL of the thigh to separate SAT from IMAT and to determine the reproducibility of parameters such as IMAT and muscle FF in various compartments of the thigh depending on the FL segmentation. Further, the effect of using the FL instead of a tight muscle envelope to quantify IMAT was evaluated in young healthy and elderly sarcopenic subjects in a retrospective analysis of a study reported earlier [23].

## Materials and methods

### Subjects and MRI scans

Two groups that had been recruited for an earlier study [23] were examined. Group 1 (G1) included 23 healthy young men (31 ± 6 years (mean value ± standard deviation), 23–46 years, BMI 23.2 ± 2.0 kg/m^2^). Group 2 (G2) included 50 elderly men with sarcopenia (77 ± 5 years, 70–86 years, BMI 26.2 ± 2.4 kg/m^2^). G1 and G2 combined, cover a wide range of muscle fat content [24].

MRI acquisition was performed using a 3T scanner (MAGNETOM Skyra^fit^, Siemens Healthineers AG, Erlangen, Germany) and an 18-channel body receive array coil. The flexible coil was wrapped around the left mid-thigh. The protocol included a clinically common T_1_ weighted (T_1_w) Turbo Spin Echo and a 6-point (6pt) Gradient Echo Volumetric Interpolated Breath-hold Examination (VIBE) Dixon sequence for chemical shift encoding-based quantification of fat and water as proton density FF. The following acquisition parameters were used: T_1_w sequence—voxel size: 0.5 × 0.5 × 3.0 mm^3^, 34 slices, matrix size: 512 × 512, TR: 844 ms, echo time (TE): 14 ms, bandwidth: 488 Hz/px, acquisition time: 2:54 min; 6pt Dixon sequence—voxel size: 0.8 × 0.8 × 3.0 mm^3^, 36 slices, matrix size: 320 × 320, TR: 14.00 ms, TEs: 1.90, 3.73, 5.56, 7.39, 9.22, and 11.05 ms, bandwidth: 710 Hz/px, flip angle: 6°, acquisition time: 1:17 min; detailed information can be found in [25]. Dixon MRI delivered proton density FF maps, which assign a percentage of fat to every voxel. Intensities in the FF maps generated by the software of the Scanner are in a range of 0–1000 corresponding to a FF of 0.0–100.0%.

To measure comparable regions of the thigh, the length of the femur and the knee joint gap was taken as a reference parameters to determine the mid-point of the femur, where the scanning volume was placed.

### Segmentation outline

**Fig. 1:**
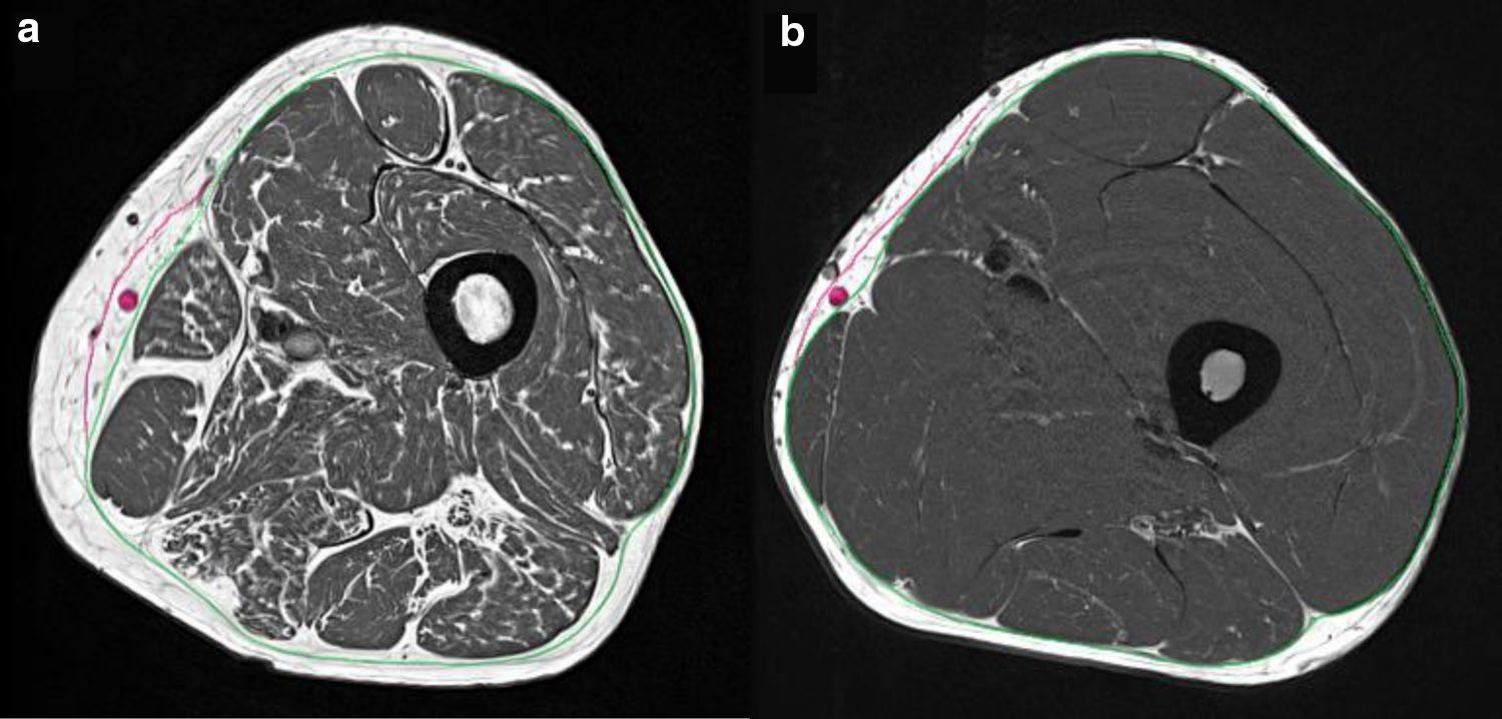
Examples of bias field corrected T_1_w images of the two subject groups. Elderly sarcopenic subject of group G2 (**a**); a healthy young subject of group G1 (**b**). The green contour indicates the position of the fascia lata (FL), the red contour indicates the fascia of the vena saphena magna (also marked in red). These two fasciae are usually highly visible but have to be distinguished

**Fig. 2:**
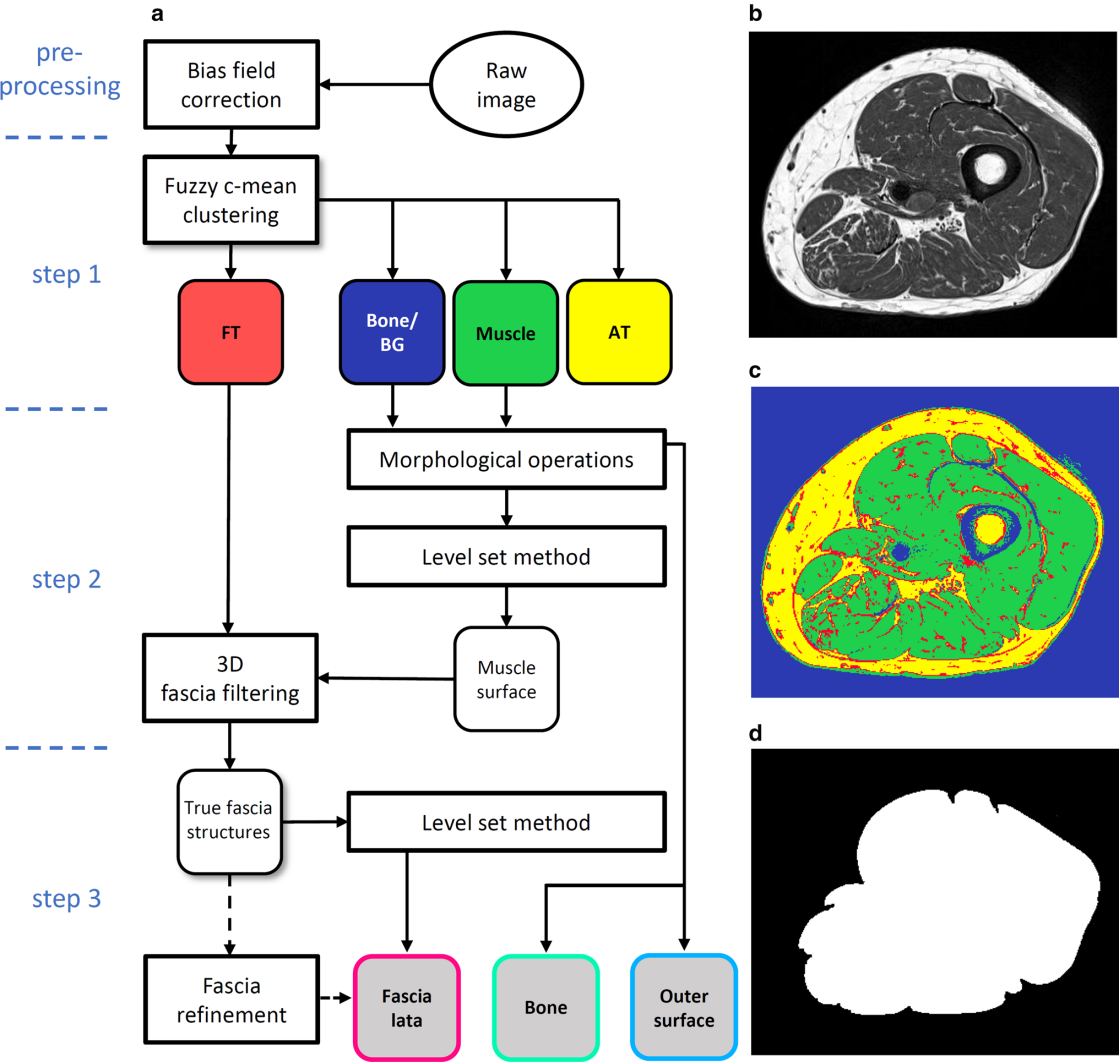
**a** Flowchart of the segmentation process. **b** Bias field corrected T_1_w image, which is the input for the fuzzy c-means clustering. **c** Result of step 1: fibrous tissue (FT, red), bone and background (BG, blue), muscle (green) and adipose tissue (AT, yellow) clusters. Sometimes veins and thicker connective tissue can also lie within the bone and BG cluster. **d** Result of step 2: tightly fitting muscle envelope

The FL segmentation was performed in the T_1_w datasets, which had high spatial resolution and good image contrast. This allowed a distinction between the FL and the saphenous fascia around the vena saphena magna [26] (Fig. 1). A flowchart of the segmentation process integrated in MIAF (Medical Image Analysis Framework, University of Erlangen) package is shown in Fig. 2a. In a pre-processing step, the N4ITK algorithm [27] was used to compensate for the bias field caused by field inhomogeneities of the scanner (Fig. 2b).

The FL segmentation process started (step 1) with a fuzzy c-means clustering that based on image intensity separated all voxels into four different clusters: muscle, AT, bone/background and fibrous tissue located at interfaces or inside the SAT (Fig. 2c). The surface of the thigh was determined from the bone and background cluster using morphological operations and a threshold filtering to remove the skin. The femur was also segmented using morphological operations.

In step 2, the level set method as described by Caselles et al. [28] was applied to the muscle cluster to find a tightly fitting envelope of the muscle ensemble of the thigh (ME: muscle envelope) (Fig. 2d). This step also excluded veins and other structures in SAT, which were included in the muscle cluster in step 1. To obtain the FL surface, a filtering process described in detail below was applied to the fibrous tissue cluster to identify FL structures in the SAT.

In step 3, a second level set process detailed in the section below combined these structures with ME obtained in step 2 to complete the segmentation of the FL. If the result of the automatic segmentation was not satisfactory, a manual refinement process could be applied.

## 3D fascia filtering

**Fig. 3:**
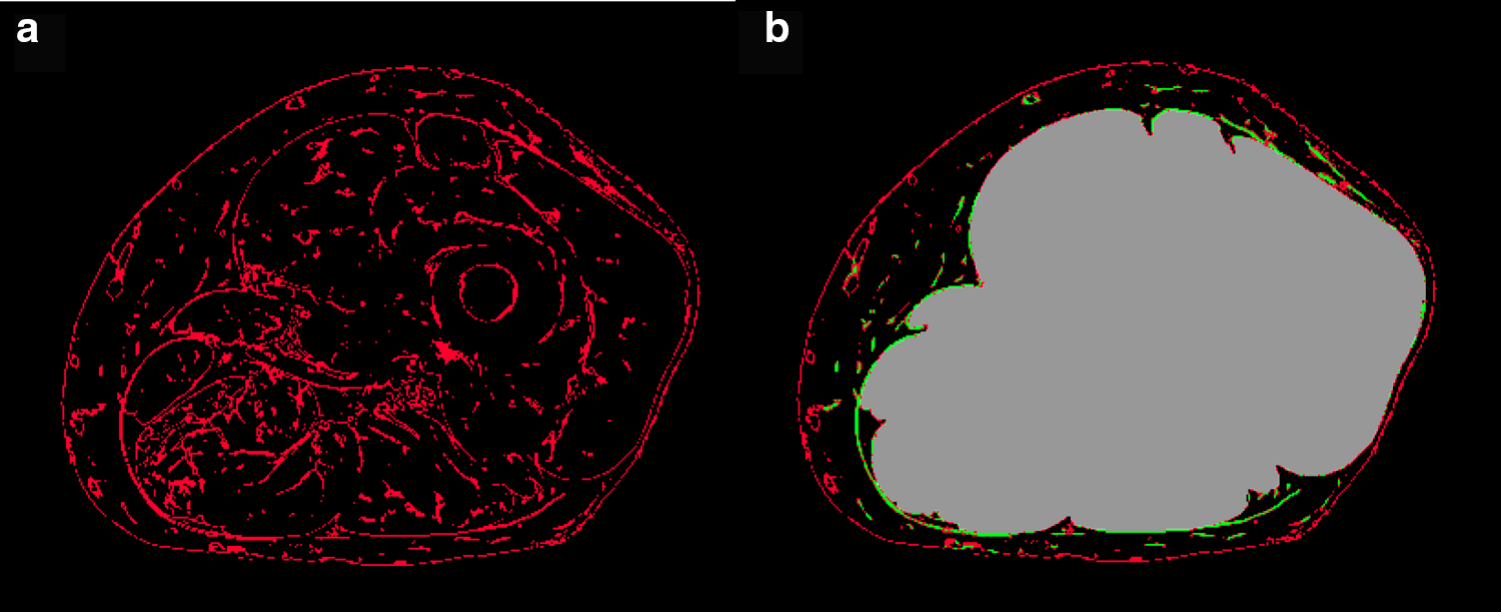
Schematic presentation of the filtering process to obtain relevant FL structures. Fibrous tissue cluster obtained from fuzzy clustering (left). Result after filtering all fibrous structures outside the muscle envelope (right). Green: 3D plate-like structures, probably part of the FL; red: structures unlikely part of the FL; for visualization only two colors, green and red are used. In reality, all voxels containing fibrous structures were continuously scaled between 0 and 1 (see text). Grey: region defined by tight muscle envelope

The fibrous tissue cluster resulting from the fuzzy c-means clustering contained several fibrous structures that were not part of the FL (Fig. 3a). These structures were typically not present in all slices of the image stack, whereas the FL is a connected 3D surface.

The undesired fibrous structures were filtered out by Frangi’s filter detection principle using the orthonormal eigenvalue decomposition of the Hessian matrix [29]. The filter distinguished between tube-like, sphere-like and plate-like shapes, the latter being of major interest for the FL segmentation. The filter computed the probability of a given fibrous structure being plate like and, therefore, considered as part of the fascia. The filter assigned a weight between 0 (did not belong to FL) and 1 (was part of FL) to all fibrous structures outside ME (Fig. 3b). A second level set segmentation starting outside ME incorporated these weighted voxels to determine the final surface defining the FL.

### Fascia refinement

**Fig. 4:**
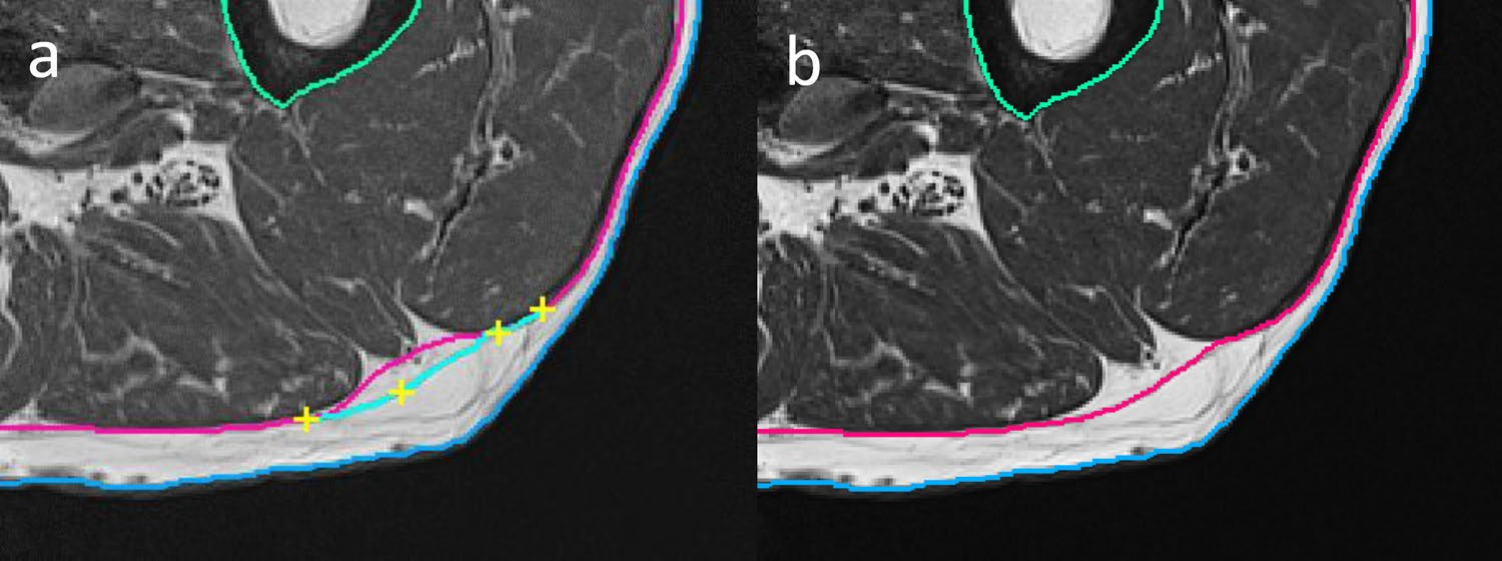
Incorrect FL segmentation in red (**a**). Application of an intelligent scissors tool using manually set seed points (yellow crosses); corrected fascia segmentation (**b**)

If the automated final FL detection failed or if artifacts interfered, a manual refinement was necessary. This was realized by a livewire approach based on the A* algorithm, a heuristic to the Dijkstra algorithm used by Mortensen’s Intelligent Scissors tool [30, 31]. Based on a lowest cost criterion, the tool found the shortest path between seed points. The cost functional considered the most distinctive edges determined from the gradient of the T_1_w images. The integration of these edges and the 3D filtered FL structures resulted in excellent performance of the manual editing tool (Fig. 4).

Once seed points were manually set in one slice, they were propagated to the next ten slices and adjusted automatically. This process favored the most probable FL location and facilitated the simultaneous correction of several slices, which significantly reduced the time required to edit the complete dataset. Basic morphological operations for additional local editing could be applied if necessary.

### IMAT and FF quantification

IMAT volume was determined in the T_1_w images by summing up all voxels of the AT cluster located in the intra-fascia (IF) VOI, i.e. the VOI enclosed by FL after excluding the femur.

**Fig. 5:**
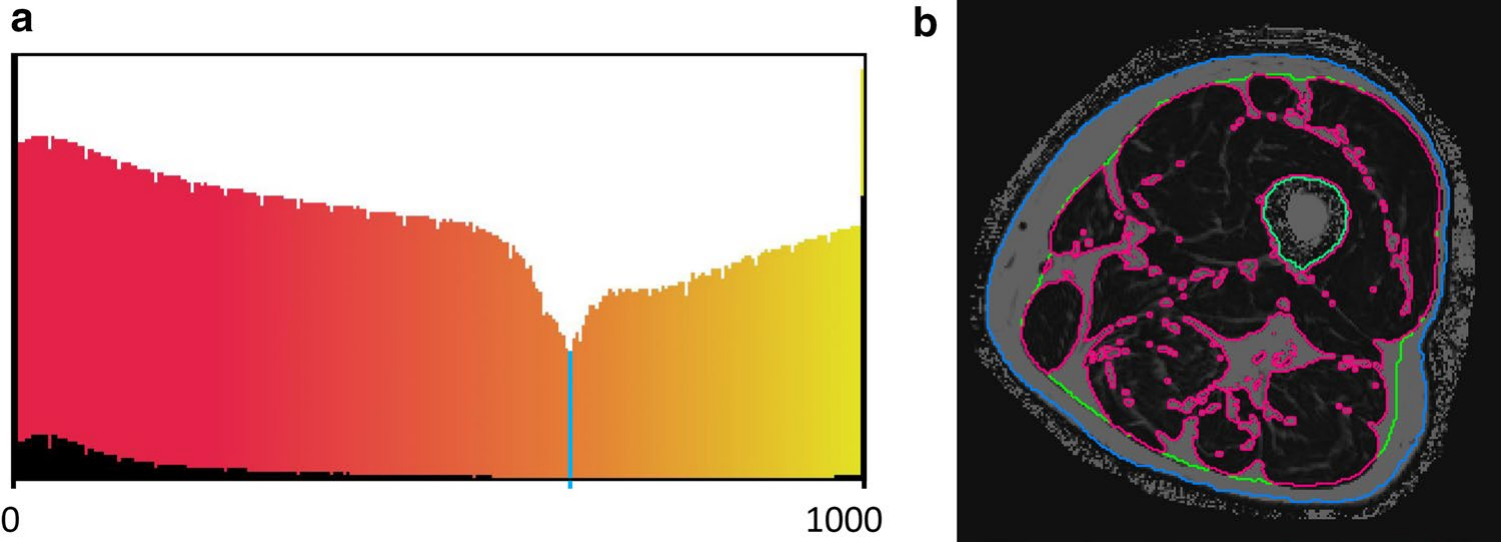
Separation of adipose and muscle tissue within the intra-fascia VOI uisng Dixon 6pt images. **a** The histograms show the grey value distribution (FF values ranging from 0 to 1000) of the whole intra-fascia VOI; the normal histogram (black) and the logarithmically scaled histogram (color gradient). The color gradient indicates muscle tissue (red) and adipose tissue (yellow). The minimum of this distribution (blue line) was used as a threshold to segment muscle tissue. **b** Segmented Dixon images; red: borders of muscle tissue (black); green: fascia lata; blue: outer surface of the thigh; the grey voxels within the FL denote IMAT

T_1_w and Dixon FF images were matched by intensity-based rigid registration of the segmented thigh VOIs. The resulting transformation matrix was then used to transform the FL segmented in the T_1_w images to the FF image stack. FF was determined in muscle tissue (MT) which was segmented using the logarithmically scaled histogram of the greyvalues within the IF VOI (Fig. 5a). The minimum of this histogram was used as threshold to separate MT form IMAT (Fig. 5b).

**Fig. 6:**
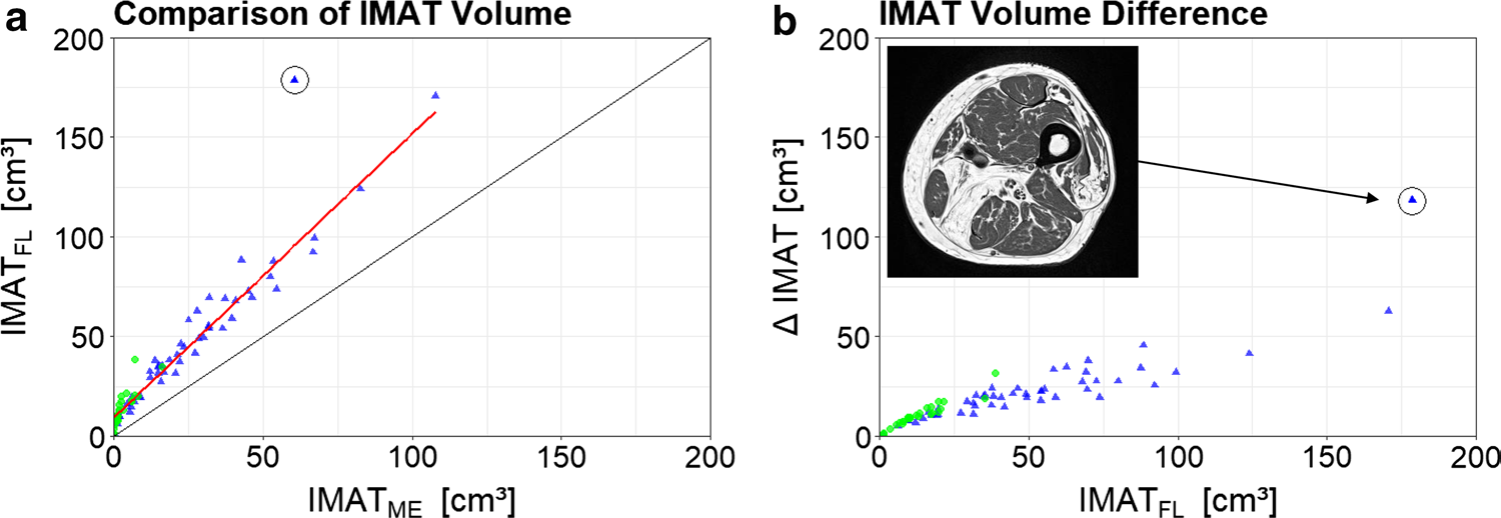
Comparison of IMAT results. **a** Correlation of IMAT volume determined by the FL (IMAT_FL_) versus the narrow muscle envelope segmentation (IMAT_ME_). **b** Absolute IMAT_FL_ values plotted against the difference of IMAT_FL_ and IMAT_ME_. Green dots represent young and blue triangles elderly subjects. One outlier is marked by a black circle. An image of this subject is shown as inset

### Statistical analysis

To examine the effect of using the FL versus a tight muscle envelope on the quantification of IMAT, the parameters ‘IMAT within the FL’ (IMAT_FL_) and ‘within ME’ (IMAT_ME_) were determined. Both parameters were compared via correlation analysis. All statistical analysis was carried out in R [32].

5 datasets randomly selected from the group of young subjects and 15 datasets randomly selected from the group of elderly subjects were used for precision analysis. Specifically, reanalysis precision was determined for volume of the IF VOI, volume of IMAT in the T_1_w scans and FF of MT (FF_MT_) in the Dixon datasets. The precision error was determined as inter-operator variability of 3 operators who each analyzed the 20 data sets once and as intra-operator variability, where 1 operator analyzed the same 20 data sets 3 times. All operators were trained by a professional physician. The first and last three slices of each dataset were omitted from the analysis due to segmentation difficulties caused by poor bias field correction. Reanalysis precision errors were calculated as the root mean square average of standard deviation (RMS-SD) in units of the measured variable and as root mean square average of the coefficient of variation (RMS-CV) in percent [33].

## Results

**Fig. 7:**
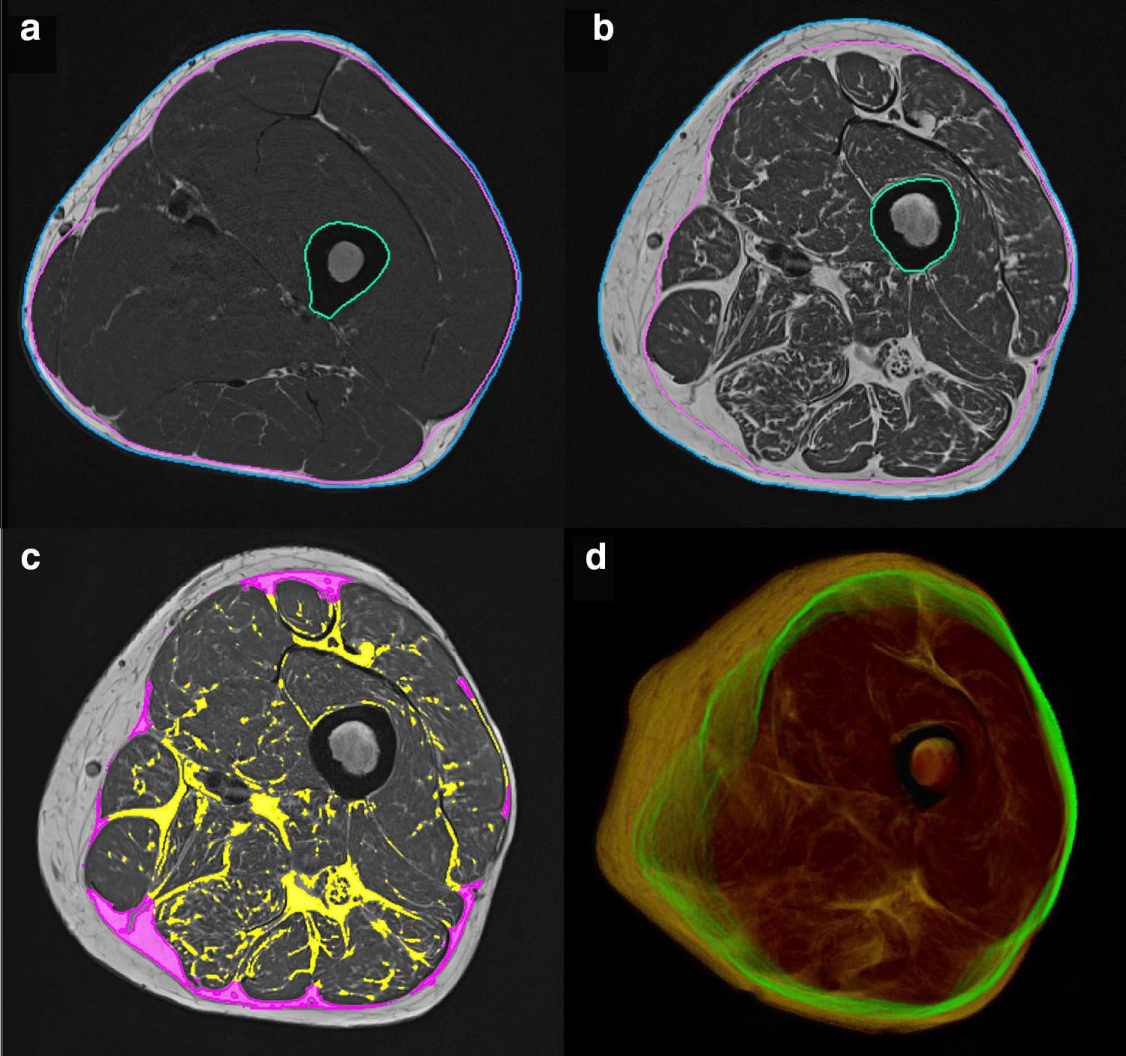
Segmentation of the T1w images of a healthy young (**a**) and an elderly (**b**) subject. Image (**c**) shows the difference in IMAT (magenta) using the segmentation of the FL versus a tight fitting envelope. **d** A 3D projection of the FL segmentation in green, with muscle in red and AT in yellow

All 73 datasets were successfully segmented and registered to the Dixon scans. Manual editing was required in the majority of datasets, but was mostly limited to fully excluding the vena saphena magna and refining the FL. In 40% of the datasets, manual corrections with the refinement tool were applied to less than ten slices and in 10% of the datasets to ten slices or more. With the livewire refinement tool, editing was applied to ten slices simultaneously; thus in practice, a very experienced operator only edited a few slices per dataset. Typical processing times including minor corrections were 1–5 min per dataset. This time increased to 8–10 min per dataset in case of major corrections. Examples are shown in Fig. 7a, b.

The registration between T_1_w and Dixon sequences was checked visually. Manual corrections were not required. Figure 7c shows an example of IMAT quantification in T_1_w data.

The Pearson correlation coefficient between IMAT_FL_ and IMAT_ME_, was *r* = 0.98. There was one outlier among the elderly subjects, which was excluded from the correlation analysis, as it showed unusually high amounts of IMAT. Figure 6 shows the correlation analysis and the outlier subject. When plotting the difference ∆IMAT (IMAT_FL_–IMAT_ME_) against IMAT_FE_, ∆IMAT showed an increase in variance with increasing values of IMAT_FE_ (Fig. 6b).

Inter- and intra-operator reanalysis precision errors for the IF VOI are summarized in Table 1. As the segmentation is easier for young than for elderly subjects, results are also shown separately for each group.

**Table 1:** Inter- and intra-operator reanalysis precision errors of G1 (*n* = 5) and G2 (*n* = 15)

	Precision errors	Inter			Intra		
		Mean	RMS-SD	RMS-CV	Mean	RMS-SD	RMS-CV
All	FF_MT_ [%]	7.29	0.02	0.04	7.30	0.01	0.02
	Volume_total_ [cm^3^]	1148	2.22	0.03	1149	0.87	0.01
	Volume_IMAT_ [cm^3^]	54.6	1.20	0.33	54.6	0.52	0.14
G1	FF_MT_ [%]	3.94	0.01	0.35	3.96	0.01	0.07
	Volume_total_ [cm^3^]	1347	2.81	0.13	1348	0.55	0.02
	Volume_IMAT_ [cm^3^]	18.1	1.75	5.80	18.0	0.25	0.82
G2	FF_MT_ [%]	8.41	0.02	0.04	8.42	0.01	0.03
	Volume_total_ [cm^3^]	1082	1.99	0.04	1083	0.95	0.02
	Volume_IMAT_ [cm^3^]	66.8	0.96	0.29	66.7	0.58	0.17

*All* G1 and G2 combined; all parameters are measured within the IF VOI, i.e. intra-fascia region, excluding the femur, *FF*_*MT*_ fat fraction of muscle tissue derived in Dixon 6pt scans by thresholding muscle and adipose tissue inside the fascia lata, *Volume*_*total*_ volume of IF derived in T_1_w data, *Volume*_*IMAT*_ intermuscular adipose tissue volume derived in T_1_w data, *RMS-SD* root mean square of the standard deviation in units of the measured variable, *RMS-CV* root mean square coefficient of variation in %

## Discussion

We presented a robust and precise semi-automatic method for segmenting the FL of the thigh muscles by combining clustering, level sets and 3D filtering of FL structures. FF_MT_ was quantified by thresholding the 6pt Dixon scans after 3D registration with the T_1_w scans. The method was applied to young healthy and elderly sarcopenic subjects.

The segmentation of the FL is difficult. Even in high-quality T_1_w MRI datasets such as those available in the current study, the FL is not always unequivocally discernible from other SAT structures. To our knowledge, no other publication has addressed this problem in depth, indeed many methods struggled with an accurate FL localization or an anatomically accurate FL definition without using manually pre-labeled images. Some disregarded the FL altogether by limiting the segmentation to individual muscles or muscle groups or even limited the segmentation to muscle tissue only. Some studies used machine learning methods to segment degenerated muscles within their epimysium [34]. However, for an accurate quantification of IMAT and SAT, the FL segmentation is a prerequisite, which in particular in elderly subjects extends beyond the tightly fitting muscle envelope of the thigh.

The FL segmentation procedure developed in this study was based on the physiological FL characteristics of forming a closed 3D surface. Hence, filtering of plate-like structures was very efficient to locate the FL components. A similar segmentation approach was reported by Kovacs et al. [35] who used a thin line filter in the SAT to find the FL, but did not exploit its closed surface property.

Despite necessary manual adjustments to fine tune the FL, the precision of the new segmentation approach was excellent. As expected, the intra-operator errors were lower than the inter-operator precision errors. Scan–rescan precision errors of a subset of the cohort of the current study has been reported earlier [25]. RMS-CV FF precision errors of the scanning process were: 2.1% for the young healthy and 1.6% for the elderly subjects, which is about ten times higher than the analysis error. Unfortunately, precision of IMAT and of FF_MT_ had not been reported in this earlier study.

The correlation analysis showed a highly linear relation between IMAT_FL_ and IMAT_ME_ across a wide range of values. The slope differed from one, resulting in an accuracy error of IMAT when using the tight muscle envelope. Thus for certain cross-sectional studies, an accurate FL segmentation may not always be necessary. However, in longitudinal studies, or even when investigating age related differences cross-sectionally it remains unclear, whether a change in IMAT can adequately be quantified, if the more peripheral parts close to the FL are neglected or whether changes in IMAT_ME_ adequately reflect changes in IMAT_FL_.

In the study cohort, there was one outlier that showed an unusually high IMAT volume. When IMAT volume in such subjects should be quantified and compared with others, an accurate FL segmentation is unavoidable. It is important to note that the discrepancies between both segmentations increased with the amount of IMAT within the subjects.

## Limitations

The quality of the segmentation approach developed in this study depends on image quality. A fundamental requirement is the detectability of fibrous tissue at the interfaces of muscles and SAT. A visual inspection of Fig. 1a shows that the voxel size of the T_1_w images of 0.5 × 0.5 × 3.0 mm^3^ fulfilled this requirement but a decrease of spatial resolution will impair the detectability of fibrous tissue and, therefore, the accuracy of the FL segmentation. The segmentation method was developed and tested on axial scans with 3 mm slice thickness. It is difficult to estimate how a larger displacement between the slices or a different orientation would affect the results. Obviously the same is true for IMAT. A decrease in spatial resolution of the T_1_w images will increase partial volume artefacts and thus decrease the precision of IMAT volume measurements.

## Outlook

A segmentation approach based on shape priors together with a statistical formulation of the level set algorithm could further automate the FL segmentation [36]. Ideally the segmentation should be fully automated, for example, using deep learning approaches [37]. However, this will require adequate training data that can easily be generated using the method developed in this study.

IMAT is the traditional parameter for the assessment of adipose tissue in T_1_w images. With the segmentation of the FL, IMAT can now be quantified. In FF Dixon images, the FL segmentation is more difficult because image contrast and often also the spatial resolution is lower compared to T_1_w images. In consequence, fibrous structures have much lower contrast in FF images. Therefore, the FL was registered to the Dixon images to determine muscle tissue FF. Theoretically, the registration step can be omitted if opposed phase Dixon images are used for the FL segmentation. In this case, probably the 3D filtering of the FL has to be improved by increasing the resolution of the Dixon images, because visual inspection also showed a better image contrast in T1w compared to opposed phase Dixon images. Based on the results of this study, the question whether for a given clinical or research setting FF of muscle tissue or IMAT volume is more relevant can now be adequately addressed.

## Conclusion

A highly precise semi-automatic segmentation method of the FL of the thigh was developed. The multi-step procedure included fuzzy c-means clustering to identify fibrous structures, Frangi’s filter detection principle to determine whether these structures were part of the FL and level set methods to determine the final FL. The combined (G1 and G2) reanalysis precision error of IMAT volume was below 0.5%. The combined reanalysis precision error of FF_MT_ was below 0.4%. The FL differs from a simple envelop of the muscle ensemble of the thigh because of additional adipose tissue between muscles and FL, in particular in elderly subjects. When quantifying IMAT in subjects with high AT infiltration, a FL segmentation is required. An approximation of the FL by a tight muscle envelope may deliver useful results in cross-sectional studies. These are important results for quantitative investigations of effects of adipose tissue due to aging, treatment and across various diseases.
